# Society Meetings

**Published:** 1908-11

**Authors:** 


### SOCIETY MEETINGS.

The Medical Society of the County of Erie held its regular meeting Monday, October 12, 1908, at 8 p.m., at the Society of Natural Science rooms, Buffalo Library Building under the presidency of Dr. Edward Clark. The program arranged by T. H. McKee, chairman of the committee on program, was carried out as follows: N. G. Russell, Cerebral hemorrhage in the newly born; A. G. Bennett, Some thoughts on sick headache; Helena Kuhlman, A case of anxiety psychosis; George N. Jack, Lymph; Allan A. Jones, Symptoms of pancreatitis; E. J. Meyer, Surgery of the pancreas.

---

The Medical Association of Central New York held its forty-first annual meeting at Buffalo, Tuesday, October 27, 1908, (postponed from October 20), under the presidency of Dr. C. C. Frederick, of this city. The papers and the proceedings will be published in subsequent issues of the Journal.

---

The Southern Surgical and Gynecological Association will hold its twenty-first annual meeting at Saint Louis, December, 15, 16, and 17, 1908. Under the presidency of Dr. F. W. Parham of New Orleans. The secretary, Dr. William D. Haggard of Nashville, is preparing an interesting program, and all members who wish to read papers should address him without delay.

---

The Mississippi Valley Medical Association, at its annual meeting held at Louisville, October 13, 14, and 15, 1908, elected officers for the ensuing year as follows:

President, J. A. Witherspoon, Nashville; first vice-president, Louis Frank, Louisville; second vice-president, Albert E. Sterne, Indianapolis; treasurer, S. C. Stanton, Chicago; secretary, Henry Enos Tuley, Louisville. The next annual meeting will be held in St. Louis, October, 1909.

---

The Buffalo Academy of Medicine held meetings during October as follows:

*Section of Pathology.*—Tuesday, October 6, at 8.30 P. M. Program. Professor Nathan Raw of Liverpool, England, delivered a paper on the relation of human and bovine tuberculosis and their treatment. Dr. Raw, one of the delegates to the International Congress on tuberculosis which met recently at Philadelphia and Washington, is one of the recognised authorities on this subject in Europe.

*Stated Meeting,* Tuesday evening, October 13, at 8.15 o'clock. The section of medicine furnished the following program. (1) Report of the commission on contagious diseases and municipal contagious hospital, Julius Ullman, chairman; (2) Contagious disease hospitals; their medical and financial importance to the community, Robert J. Wilson, superintendent of contagious hospitals, department of health, New York city; (3) A brief demonstration of the manner contagious disease records are kept by the Buffalo department of health, Franklin C. Gram.

*Section of Surgery.*—Tuesday evening, October 20, at 8:30 o'clock. Program: Symposium on gonorrhea. (1) Morphology of the gonococcus with its differentiation by staining from other urethral bacteria, A. A. Thibaudeau; (2) The treatment of acute gonorrheal urethritis in the male, David E. Wheeler; (3) The treatment of chronic gonorrheal urethritis and its complications in the male, Nelson W. Wilson; (4) The treatment of gonorrhea in the female, Earl P. Lothrop; (5) Prophylaxis of gonorrhea, James A. Gardner.

*Special Meeting,* October 27, 1908, at 8.30 P. M. Council Meeting at 8.15 P. M. Order of business for academy:

(a) Balloting on new members; (b) Consideration of question of incorporation as announced by former notice of this meeting dated September 23; (c) The committee on permanent home submitted several propositions with plans and estimated cost. The section of obstetrics and gynecology provided the following program. Ectopic pregnancy, Herman E. Hayd, M. D.

